# A Computational Model of a Descending Mechanosensory Pathway Involved in Active Tactile Sensing

**DOI:** 10.1371/journal.pcbi.1004263

**Published:** 2015-07-09

**Authors:** Jan M. Ache, Volker Dürr

**Affiliations:** 1 Department of Biological Cybernetics, Faculty of Biology, Bielefeld University, Bielefeld, Germany; 2 Cognitive Interaction Technology–Center of Excellence, Bielefeld University, Bielefeld, Germany; Imperial College London, UNITED KINGDOM

## Abstract

Many animals, including humans, rely on active tactile sensing to explore the environment and negotiate obstacles, especially in the dark. Here, we model a descending neural pathway that mediates short-latency proprioceptive information from a tactile sensor on the head to thoracic neural networks. We studied the nocturnal stick insect *Carausius morosus*, a model organism for the study of adaptive locomotion, including tactually mediated reaching movements. Like mammals, insects need to move their tactile sensors for probing the environment. Cues about sensor position and motion are therefore crucial for the spatial localization of tactile contacts and the coordination of fast, adaptive motor responses. Our model explains how proprioceptive information about motion and position of the antennae, the main tactile sensors in insects, can be encoded by a single type of mechanosensory afferents. Moreover, it explains how this information is integrated and mediated to thoracic neural networks by a diverse population of descending interneurons (DINs). First, we quantified responses of a DIN population to changes in antennal position, motion and direction of movement. Using principal component (PC) analysis, we find that only two PCs account for a large fraction of the variance in the DIN response properties. We call the two-dimensional space spanned by these PCs ‘coding-space’ because it captures essential features of the entire DIN population. Second, we model the mechanoreceptive input elements of this descending pathway, a population of proprioceptive mechanosensory hairs monitoring deflection of the antennal joints. Finally, we propose a computational framework that can model the response properties of all important DIN types, using the hair field model as its only input. This DIN model is validated by comparison of tuning characteristics, and by mapping the modelled neurons into the two-dimensional coding-space of the real DIN population. This reveals the versatility of the framework for modelling a complete descending neural pathway.

## Author Summary

Many nocturnal and burrowing animals rely on their tactile sense to explore the surrounding space, and tactile cues are often used to adapt locomotion to a structurally complex environment. Most mammals use facial whiskers for active tactile exploration, while most insects use their antennae. Since whiskers and antennae are long, thin, cylindrical structures, they must be moved to probe the surrounding space. The nervous system therefore has to keep track of tactile sensor movement by encoding sensor position and motion in order to locate tactile contacts. Here, we model a descending neural pathway of the stick insect, which transfers information about tactile sensor movement to thoracic neural networks with short latency. We show that information about sensor position and motion can be derived from a single class of proprioceptors at the antennal joints, and present a computational model that explains the activity of four previously described groups of descending interneurons during antennal stimulation. Our model is validated against electrophysiological data on antennal mechanoreceptors and descending interneurons.

## Introduction

Active tactile sensing is a widespread strategy for near-range orientation in the animal kingdom. The whiskers of mammals [1] or antennae of insects and crustacea [2] are moved actively to acquire information about the near-range environment. Tactually elicited changes in behaviour often involve fast, adaptive motor reactions. In insects, these adaptive movements include rapid body axis inclination [3] or turning [4], but also aimed reaching movements of the front legs [5]. These behaviours have in common that tactile information must be mediated to thoracic locomotor networks with short latency.

To date, several descending interneurons (DINs) likely to be involved in tactually induced adaptive behaviours have been characterised (e.g., [6,7]). As yet, very little is known about how the population of descending neurons encodes and mediates the sensory essentials to lower motor control centres that drive leg movement. Current modelling approaches to descending sensory-motor control concern detailed models of individual descending neurons (e.g., [8]), or conceptual models of how a population of neurons with similar response properties may encode complex information such as object motion [9]. Yet other models follow a control-theoretic approach devoid of any neuronal components (e.g., [10]) or use analogue signals to simulate the bulk activity in a descending pathway [11], thus not taking into account the diversity of descending interneurons involved. Missing until today is a computational framework that allows both the simulation of individual known neurons, and the combined modelling of an entire population of descending neurons, while accounting for diverse coding properties.

Here, we propose such a computational framework for modelling a population of DINs and their afferent sensory input. The DIN models simulate the multivariate information transfer from an active touch system to a motor control system. For three reasons, we choose the antennal mechanosensitive pathway of the stick insect *Carausius morosus* as the paragon for our model: i) A behavioural reach-to-grasp paradigm in stick insects [5] requires fast information transfer of antennal posture and movement; ii) both the antennal tactile system [12,13] and the thoracic leg motor control networks [14,15] are well studied; and iii) a population of spiking DINs has been described that conveys diverse, multivariate information about antennal posture and movement to thoracic neural networks [16]; [17]. Our objective was to devise models of several different DIN types, including position- and motion-sensitive DINs, drawing from a small set of computational modules such as linear filters and a noisy spike generator.

We include a model of the afferent input that drives these DINs. This "afferent model" is upstream from the DIN models and simulates the afferent activity generated by antennal hair fields. Hair fields are insect-specific patches of external mechanoreceptors which, because of their location, function as proprioceptors. Our model simulates known properties of antennal hair field afferents, which can encode antennal joint angle and angular velocity owing to their phasic-tonic response characteristics [18–20]. The objective of the afferent model is to test how much of the DIN response properties can be explained by a single type of mechanoreceptor afferents.

Our model explains spike response patterns of four distinct DIN classes as described by [16], making it an exceptionally complete and versatile model of a descending neural pathway. We show that input from hair fields alone is sufficient to explain a large fraction of the response variants found in a real DIN population, including position-and motion-sensitive, ON-type, and OFF-type interneurons. For validation, we present a data-driven analysis of the multivariate DIN coding properties measured in electrophysiological experiments. By doing so, we derive a two-dimensional coding-space and show that real and modelled neurons are co-localised in this coding-space. Thus, we present a model that can explain the activity of an entire population of descending interneurons involved in the control of adaptive locomotion.

## Material and Methods

### Experimental data for model validation

Experimental data for model validation and derivation of the coding-space (see below) were taken from a recent electrophysiological study on descending interneurons (DINs) in the stick insect (*Carausius morosus*) [16]. We used a sub-sample of 59 intracellular DIN recordings. All of these DINs were sensitive to changes in antennal position or motion. Stimulation of the antenna was delivered by a custom-built stimulator that moved only one of the two antennal joints, the scape-pedicel joint (Sc-Pd joint). The stimulator deflected the pedicel and the base of the flagellum in a contact free manner, by moving a magnet that guided a metal minutin pin that was inserted into the cut flagellum. The proximal antennal joint, the head-scape joint, was kept immobile, making sure that only the Sc-Pd joint was deflected. Thus, we predominantly stimulated proprioceptors of the Sc-Pd joint. Additional slight bending of the flagellar base and associated stimulation of cuticular strain sensors (campaniform sensilla) could not be excluded (see discussion, and [16]). For further details about the stick insect antennal sensory and motor system, see [13].

**Fig 1:**
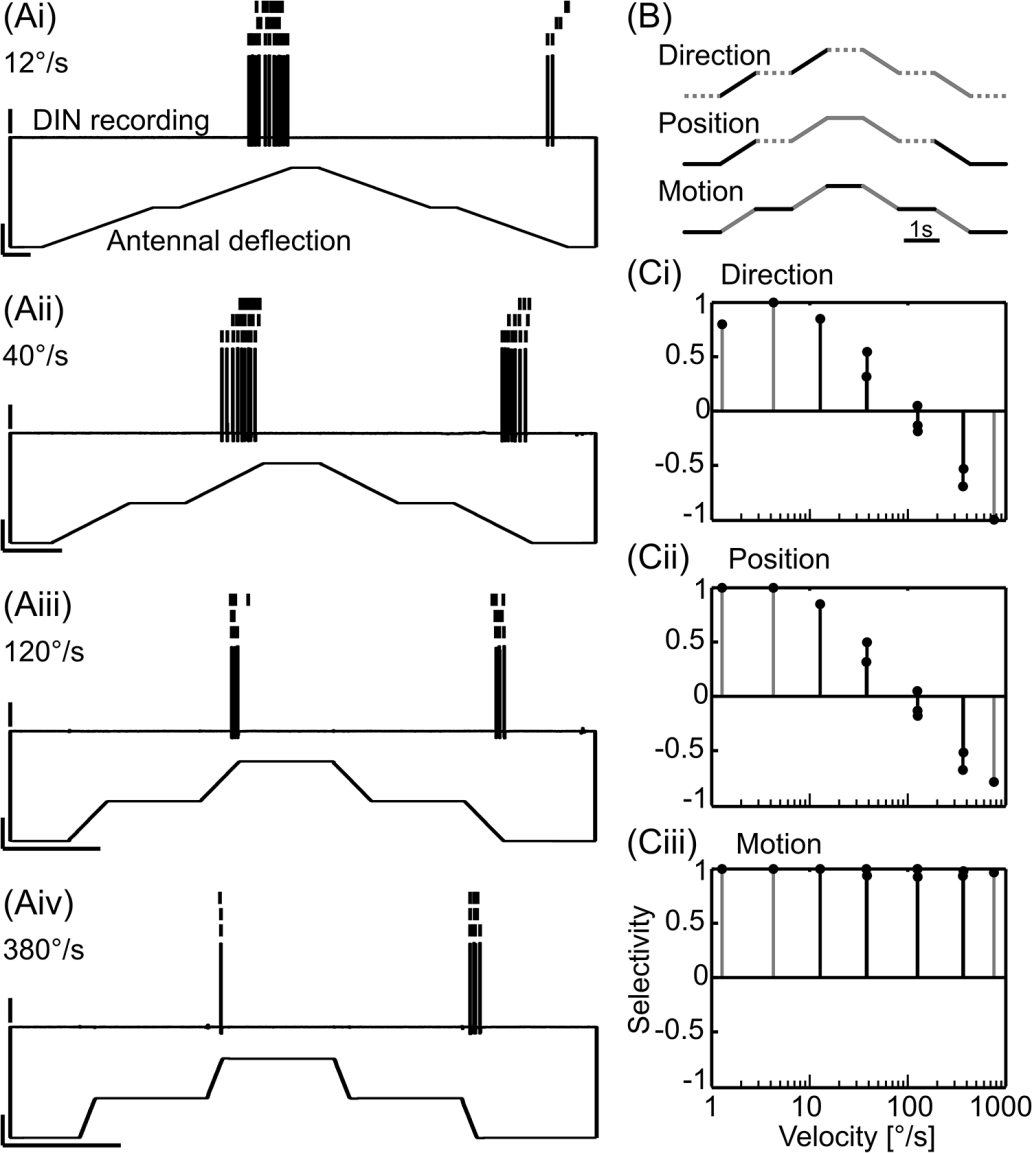
Derivation of selectivity scores. Three selectivity scores were used to indicate the ability of an individual DIN to mediate information about direction, position and motion of the antenna to thoracic neural networks. A) Representative spike response to staircase stimuli with identical amplitude but different velocities (i-iv). Scale bars: 20 mV, 40°, 1s. Lower traces, antennal Sc-Pd joint angle; upper traces, intracellular DIN recording; vertical lines, spikes during three sweeps of subsequent identical stimuli, lowest trace is the same as the original recording. B: Schematics show which parts of the stimulus intervals were used to calculate the selectivity scores. The mean spike rates during the black parts of the stimulus interval were contrasted against the mean spike rates during the grey parts (see Equation 1). Dotted parts were not considered for the given selectivity score. C) Graphs show the selectivity of the DIN in A) for the three parameters (Ci, direction; Cii, position; Ciii, motion) at different stimulus velocities. The mean selectivity scores with a black line were used for the subsequent PCA.

The stimulus time course was a staircase of ramp-and-hold deflections. Each trial comprised two upward and two downward ramps (i.e., antennal movement) separated by hold phases without antennal movement (Fig 1A). In each trial, the Sc-Pd joint was initially held at the ventral extreme position (-50°), then moved to the resting position (0°), and further to the dorsal extreme position (50°). After the hold phase at the dorsal position, the antenna was moved back in the reverse sequence. Therefore, the stimulus time course per trial was symmetrical in space and time. Between trials, the deflection velocity was varied between 1 and 800°/s. Trials were repeated two to eight times, then the ramp velocity was changed pseudorandomly. For each DIN, stimulus trials covered a wide range of naturally occurring movement velocities.

For further analysis of the spike response of each DIN, membrane potential traces were resampled at 5000 Hz and imported to Matlab (Version 7.9, Mathworks, Natick, USA). All data analysis and modelling was done in Matlab, using custom-written scripts. For a detailed description of the electrophysiological methods, see [16].

For validation of the "afferent model", we used a graphical comparison with published data on antennal hair field responses in the cockroach [20].

### Mapping of model output into the coding-space of real DINs

In order to allow a quantitative comparison of the physiological coding properties of the real DINs with the output of our DIN model, we needed to define an appropriate frame of reference. This was done in two steps.

In the first step, we used three major descriptors of the stimulus-induced spike responses of a recorded DIN, and determined the value of each descriptor at four different stimulus velocities. The descriptors were scores of the direction, position, and motion selectivity of the DIN response. As selectivity scores, we calculated three signed contrast measures relating the mean spike rates of the DIN during certain parts of the ramp-and-hold stimulus, using the equation:

$$s=\frac{A-B}{A+B}$$

where s is the seletivity score, and A and B are the mean spike rates of a given DIN during the stimulus parts depicted in the schematics in Fig 1B (part A: black solid lines; part B: grey solid lines). Thus, s varied continuously between 1 (A > B) and -1 (B > A). Rather than using the absolute value of s, we deliberately kept the sign at this point of the analysis, because distinct groups of DINs have ON- or OFF-properties [16] and, therefore, differ in the sign of s but not necessarily in its absolute magnitude. Larger absolute values of s indicated stronger selectivity. Direction selectivity was scored by relating the mean spike rate during the upward stimulus ramps (levation of the antennal Sc-Pd joint) to the mean spike rate during the downward stimulus ramps (depression of the antennal Sc-Pd joint, top schematic in Fig 1B). As a result, direction selectivity was 1 if the DIN spiked only during levation, and -1 if the DIN spiked only during depression of the Sc-Pd joint. For scoring the position selectivity, the mean spike rate during the lower (ventral) stimulus ramps and hold phase was contrasted against the mean spike rate during the upper (dorsal) stimulus ramps and hold phase (middle schematic in Fig 1B). Finally, motion selectivity was scored by relating the mean spike rate during antennal movement (stimulus ramps) against the mean spike rate during rest (hold phases, lower schematic in Fig 1B). In case of several trials with repeated identical stimuli, spike rates were averaged across trials. Generally, the variability in the response to such repeated stimulus presentations was low, which can be seen by similar scores for any given velocity in Fig 1C. This indicated that stimulus history was of little importance.

For example, the DIN in Fig 1 responded strongly to Sc-Pd joint movement towards the dorsal and ventral extreme positions (Fig 1Ai-iv). Moreover, the number of spikes and the mean spike rate depended strongly on the stimulus velocity. Increasingly faster upward movement resulted in fewer spikes at lower rates, while increasingly faster downward movement resulted in higher spike rates. (Fig 1Ai-iv). This resulted in velocity-dependent direction and position selectivities (Fig 1Ci and 1Cii) but nearly constant motion selectivity (Fig 1Ciii). The motion selectivity was always approximately 1 because the DIN almost never spiked during hold phases.

Since the coding properties of each individual DIN were quantified by only three types of descriptors, the analysis was not sensitive to more particular properties of some DINs. For example, the selectivity scores did not include a measure of relative direction, so that a DIN like the one shown in Fig 1 got pooled with other motion-selective neurons, despite the fact that it spiked only during movement away from the resting position. In the case of the relative direction selectivity, this didn't matter much, because only two DINs of the type shown in Fig 1 were included in the analysis. Nevertheless, this example illustrates that the versatility of our analysis framework comes at the cost of losing detail in the description of DIN types with rare and particular properties. Despite this trade-off, the selectivity scores gave a reasonably detailed account of the most common DIN response properties, and allowed us to characterize the overall stimulus encoding properties of each DIN by a small set of selectivity scores (compare Fig 1A and 1C). We used the selectivities at four intermediate velocities for further analyses (Fig 1C, black symbols), which reduced the number of variables that described each DIN to 12.

**Fig 2:**
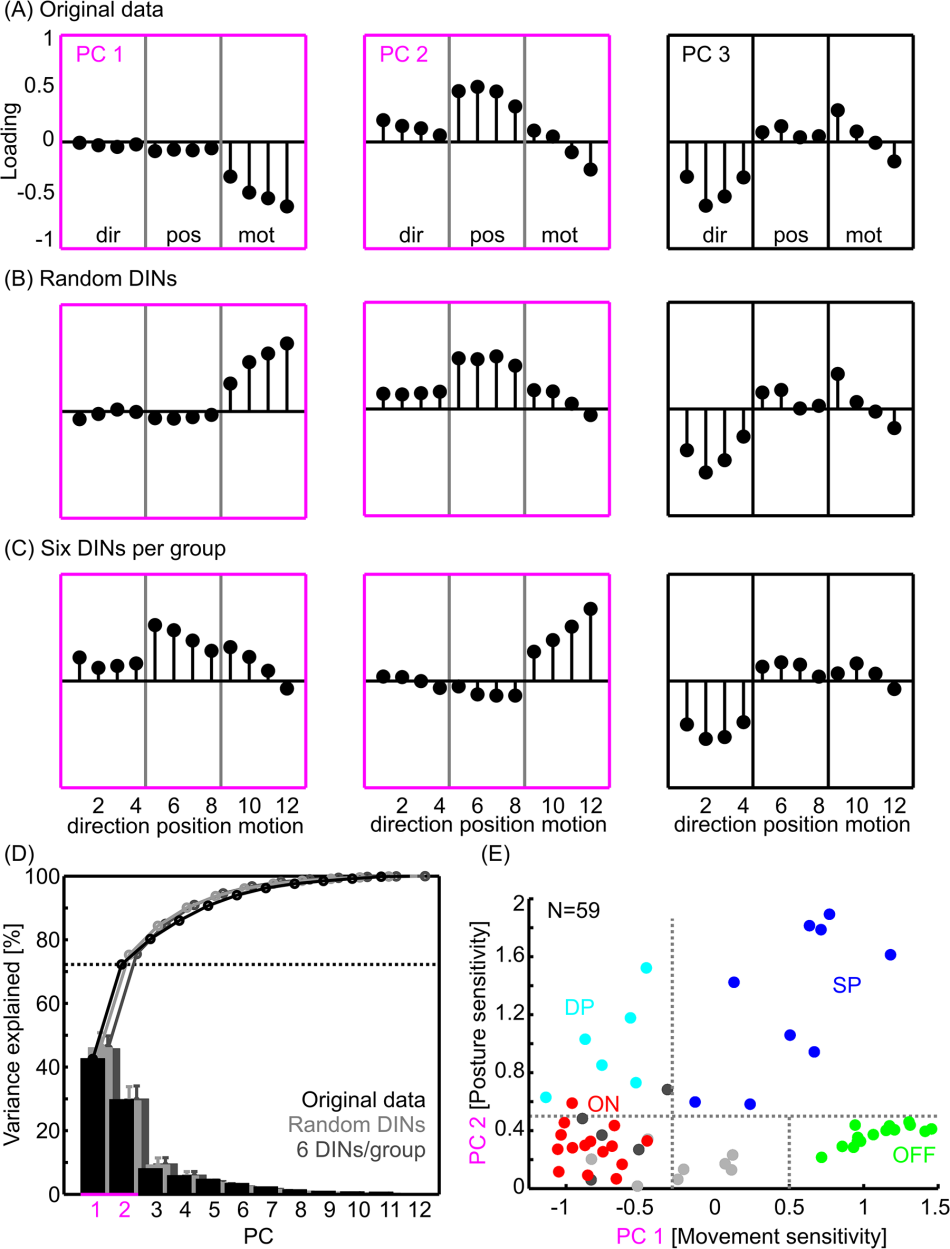
Principal Component Analysis of the DIN selectivities. A) PCs of the original dataset (N = 59 intracellularly recorded DINs). B) PCs of a sample of 50 DINs, drawn randomly with replacement from the complete sample. C) PCs of a sample of 30 selected DINs from which 50 were drawn randomly with replacement. N = 6 DINs were selected from each of five empirically defined groups (simple position-sensitive, dynamic position-sensitive, OFF-type velocity-sensitive, ON-type velocity-sensitive, and unspecific movement-sensitive DINs). Out of this sample of 30 neurons, 50 were randomly selected with replacement to calculate the PCA. D) Variance explained by the principal components. Black: Original data, N = 59 neurons. Light grey: randomly sampled neurons. Dark grey: six DINs per group. For the latter two, results were averaged across 100 random samples. The standard deviation across these samples was small, indicating that the variance explained was little affected by a sampling bias. The dotted horizontal line indicates the variance explained by the first two PCs for the original dataset (marked in magenta throughout). E) 59 DINs in the coding-space, as defined by the first two PCs of the original dataset in A. The y-axis shows the absolute PC 2 score, i.e., irrespective of dorsal or ventral joint angles. Each DIN was colour-coded according to the empirically determined group it belongs to: Cyan, dynamic position-sensitive DINs (DP); red, ON-type velocity-sensitive DINs (ON); blue, simple position-sensitive DINs (SP); green, OFF-type velocity-sensitive DINs (OFF); dark grey, few-fast type DINs, light grey, unspecific movement-sensitive DINs. Grey dotted lines indicate linear thresholds that were sufficient to separate the important DIN groups with high accuracy.

In the second step of the coding-space analysis, we applied multivariate statistics analysis to further reduce the dimensionality of the mapping. More specifically, we applied a principal component analysis (PCA) in order to transform the twelve-dimensional space spanned by the selectivity scores into an orthogonal 12-D space in which the axes pointed into the directions that explained most of the data variance, i.e., the principal components (PC). Each PC must be viewed as a linear combination of twelve selectivity scores. Because in PCA the eigenvalue of each PC reports the fraction of the total data variance explained, we could determine those PCs which explained more variance than each selectivity score alone (Kaiser-Guttmann criterion). In our case, this was the case for two PCs only (see Fig 2 and Results section). Throughout this study, we will call the space spanned by the first two PCs the "coding-space" of the DIN population. Since PC1 loaded mostly on motion selectivity, and PC2 loaded mostly on position selectivity, we chose to label the axes of the coding space ‘movement sensitivity’ (PC1) and ‘posture sensitivity’ (PC2, see Results).

The same analysis as described for the real neurons above was applied to the modelled neurons, allowing us to validate the model against experimental data, and to conduct a sensitivity analysis of model parameters.

### Model components

The computational framework devised in this study comprises two types of models: an "afferent model" capturing the essential properties of antennal hair field afferents, and a "DIN model" with four variants, capturing the essential response properties of different DIN types. The afferent model is upstream to the DIN model variants. For both models, we used the same computational modules, such as gain factors, thresholds, linear filters, and a noisy spike generator. These modules were deliberately kept at a simple, conceptual level, to not make more assumptions than justified through experimental data. The response properties of hair field afferents [20] and DINs [16], which we chose for model validation, were described by means of their spike response patterns. Therefore, it was essential to ground our model validation on spike rate comparisons.

Time-varying data was filtered using linear first-order high- and low-pass filters with the time constant (tau) as the only variable. Series of filters were used to convert stimuli into appropriate activation functions that may be considered an approximation of a neuron’s membrane potential.

Using these activation functions, a noisy spike generator yielded the spike patterns of the modelled neurons, both in hair field afferents and DINs. A spike was generated at a given time step, *t*, if the scaled activation function, *act(t*) exceeded a random number, *rand*, drawn from a uniform distribution between zero and one:

spike(t)=1ifact(t)*dS*Rmax≥rand;otherwisespike(t)=0,

where *spike(t)* is a discrete-valued time series indicating whether or not a spike is being fired at time *t*, *dS* is the time step, and *Rmax* determines the spike rate. Appropriate choices of *dS* and *Rmax* thus allowed scaling the mean spike rate of the modelled neuron to that observed in a real neuron. As dS was kept fixed at 1 ms, only *Rmax* was adjusted. The spike generator is probabilistic, and the likelihood of the modelled neuron to spike is determined by the activation function and *Rmax*. Spikes cannot be elicited at activation levels below zero. For activation levels between zero and one, the likelihood of a spike to be elicited at a given *Rmax* rises linearly.

The noisy spike generator was thus appropriate for turning continuous activation functions into discrete spike times, as it generated relatively broad spike rate distributions around the firing rate *Rmax*. However, the spike generator was not designed to generate exact spike times for a particular type of neuron. The simplicity of the spike-generating mechanism permitted us to use it for the mechanosensory afferents as well as for all DIN types. Further details and model parameters are explained in the Results section.

## Results

### Mapping the descending interneuron population into a multivariate coding-space

Our first objective was to quantify the activity of a population of recorded antennal mechanosensory DINs as the basis for our model. We chose three main descriptors to distinguish between episodes of antennal stimulation. These were: i) movement *versus* rest, ii) upper *versus* lower working-range, and iii) upward *versus* downward direction of movement (Fig 1B). Accordingly, we used three selectivity scores (motion, position and direction) to map the spike response pattern of each DIN into a three-dimesional coordinate system. Since antennal movement velocity clearly modulated the responses of several DIN types ([16], see example shown in Fig 1), we included selectivity scores determined at four different movement velocities. Thus, each DIN was characterized by a vector of twelve values comprising three types of selectivity scores for each one of four joint angle velocities. Joint angle velocities ranged between 12 and 400°/s, covering a large part of the natural velocity range during tactile searching and sampling.

A total of 59 DINs were included in the analysis. To reduce the dimensionality of the original dataset, we used principal component analysis (PCA). This served two major purposes: first it allowed us to validate our earlier, inspection-based classification scheme [16] by a data-driven method; second, it allowed us to explain a substantial part of the variance in the coding-space of the DINs, using a minimal set of orthogonal axes. The number of meaningful axes was determined by use of the Kaiser-Guttman criterion. This criterion includes only principal components (PC), i.e., axes of the coding-space, which explain a larger fraction of the total variance than any of the twelve original variables would (1/12 = 8.3%).

According to this criterion, only the first two principal components (PCs) were meaningful. Together, they explained about 73% of the variance in the selectivity scores (Fig 2D, black dotted line). PC1 loaded most strongly on motion selectivity (Fig 2A, PC1) and explained 43% of the total variance in the selectivity scores (Fig 2D). The loading of motion selectivity scores gradually increased with increasing velocity, indicating that there was a tendency for stronger motion selectivity during faster displacement (Fig 2A, PC1). The ‘loading’ is a scaling factor that indicates the weight of a given score for a PC. Note that the sign of the loading is not meaningful, because the direction of the axis is arbitrary. PC2 loaded predominantly on position selectivity (Fig 2A, PC2), with a weak but graded influence of both direction and motion. Three of the four position selectivity scores had about equal weight, indicating similar position selectivity at all but the fastest velocities. PC2 explained about 30% of the total variance (Fig 2D, PC2). The third PC loaded most strongly on direction selectivity, with hardly any influence of position and a weak, graded, and clearly bidirectional influence of motion (Fig 2A, PC3). The latter indicates opposite effects of very slow and very fast velocities. Since PC3 explained less than eight percent of the total variance (Fig 2D), we did not include it in the further analysis. In summary, the population of DINs was most sensitive to Sc-Pd joint movement and the Sc-Pd joint angle, i.e., pointing direction, of the antenna. In contrast, direction selectivity explained only little variance in the data, suggesting that it is not a key variable of the coding-space of these neurons.

To make sure that these results were not influenced by a sampling bias due to different numbers of recordings obtained from different DIN types, the PCA was repeated on random samples (Fig 2B) and on selected subsamples containing equal numbers of DINs from the different, empirically defined groups (Fig 2C). Grouping was based on the criteria presented in [16]. The PCAs on both random and selected DINs yielded very similar results as the original PCA on the full dataset (Fig 2A, 2B and 2C). Whereas the absolute loadings of the first three PCs were always similar, the order of PCs one and two was swapped in some subsamples (compare, e.g., Fig 2A PC2 and 2C PC1), and the direction of the PCs was inverted in some cases (compare, e.g., Fig 2A PC1 and 2B PC1). Both the order and the direction of the PCs are not important to our analysis, because they only affect the arrangement and direction of the axes of an orthogonal subspace of the original data. Since the loadings were essentially the same, the orientations of the axes that span this subspace varied only little. Moreover, the variance explained by the first three PCs was similar for all three PCAs, with slightly more variance explained in case of the random samples than in case of the original data. This result varied little when using different random subsamples (Fig 2D, error bars). We conclude that our data-driven construction of a two-dimensional (2-D) coding-space of DIN responses was not influenced by a sampling bias. The two significant axes of this coding-space essentially correspond to antennal posture and movement sensitivity, with very little effect of movement direction. Accordingly, we conclude that our set of DINs mainly mediates position and motion information to thoracic neural networks.

Within the 2-D coding-space (PC space) derived above, we plotted the 59 DINs according to the projection of their 12 selectivity scores onto PCs 1 and 2 (Fig 2E). With regard to the type of selectivity score that loaded most strongly on PC1 and PC2, we chose to label the axes of the coding-space ‘movement sensitivity’ (PC1) and ‘posture sensitivity’ (PC2).

As a result, each DIN’s sensitivity to antennal movement (PC1) and posture (PC2) is intuitively understandable by its location in coding-space. Note, however, that PC1 and PC2 are strongly correlated but not equal to the selectivity scores for ‘motion’ and ‘position’, and that a DIN's location in coding-space does not provide a direct measure of its sensitivity.

To further simplify the interpretation of the data, the negative part of PC2, i.e., the part corresponding to ventral joint angles, was flipped over, such that only the absolute PC2 score, or posture sensitivity, is shown. As a consequence, DINs at the top of the graph responded most strongly to strong excursion of the Sc-Pd joint (either dorsal or ventral), whereas the response of DINs at the bottom of the graph exhibited little influence of the joint angle. DINs in the far positive range of PC1 fired more during rest (stimulus hold phases) than during movement (stimulus ramps), whereas DINs in the middle and negative range fired more during movement than during rest (Fig 2E).

### Empirical DIN groups can be linearly separated in the coding-space

The distribution of DINs within the coding-space was widely spread (Fig 2E), showing that recorded DINs had very different selectivity profiles. Although not all DINs fell into clear clusters, it was possible to assign them all to distinct groups (Fig 2E). This was done in two ways.

First, we manually assigned each DIN to one of six groups after inspection of the original recording traces, according to empirically defined criteria [16]. The criteria were the baseline spike rate, spike patterns during antennal stimulation, sensitivity to antennal movement velocity, position-sensitivity, peak spike rates, and response latencies. In a second step, we attempted to define linear separators that divide the coding-space into distinct sub-regions in a functionally meaningful manner.

In the first approach, the data point of each DIN was coloured according to the group it was assigned to (Fig 2E). The major DIN groups were *simple position-sensitive DINs* (dark blue, SP), which spiked whenever the Sc-Pd joint angle was within a certain range; *dynamic position-sensitive DINs* (cyan, DP), which spiked during antennal movement within a given joint angle range; *ON-type velocity-sensitive DINs* (red, ON), which spiked during antennal movement; and *OFF-type velocity-sensitive DINs* (green, OFF), which had a high baseline spike rate and spiked less during antennal movement (see [16], for a more detailed description of the DIN response properties). The data-set also contained DINs of the *few-fast type*, which fired one to two spikes during fast Sc-Pd joint movement only (dark grey), and *unspecific movement-sensitive DINs*, which had a high background spike rate and fired few additional spikes during fast antennal movement (light grey).

As can be seen in Fig 2E, each symbol colour is confined to a sub-region of the coding-space. Indeed, it was possible to separate all but one DINs of the four major groups into disjunct regions of the coding-space using simple linear thresholds (Fig 2E, grey dotted lines). The right vertical line separates DINs that fired more during movement from those that fired more during rest, and the horizontal line separates position-sensitive DINs from the other groups. Only one DIN of the ON group (red) had a posture sensitivity that fell into the range of the position-sensitive groups DP and SP (light and dark blue, respectively). This corresponds to 98% of correct class assignments. Therefore, the data-driven analysis allows automatic, thus unbiased classification of DINs with high precision.

We conclude that, despite the fact that the 2-D coding-space was constructed in a data-driven way, linearly separated sub-regions of this coding-space coincided very well with the empirical DIN groups proposed earlier. For example, *ON-type velocity-sensitive DINs* formed a tight cluster in the lower left corner of the coding-space, i.e., at high movement- and low posture sensitivity (red dots, ON, Fig 2E). The relatively tight clustering indicates low variability in the *ON-type velocity-sensitive DIN* population, and thus might suggest that relatively few individual DINs belong to that cluster. At the opposite end of the movement sensitivity range, *OFF-type velocity-sensitive DINs* formed a tight, stripe-shaped cluster that spanned regions from high movement sensitivity to low movement sensitivity, close to the centre of the antennal coding-space (green dots, OFF, Fig 2E). As expected, all *OFF-type velocity-sensitive DINs* fired more during hold phases than during movement, but their sensitivity to antennal movement varied strongly.

Position-sensitive DINs did not form very tight clusters, but occupied distinct regions in coding-space (blue and cyan dots, Fig 2E). *Simple position-sensitive DINs* (SP) occupied the upper right half of the coding-space, indicating that they fired less during movement than an average DIN. They were still located to the left of OFF-type velocity-sensitive DINs, indicating that their spike rate was unaffected by antennal movement. *Dynamic position-sensitive DINs* (DP) spiked almost exclusively during antennal movement, and accordingly covered the same region as ON-type velocity-sensitive DINs on PC1 (cyan dots, Fig 2E), but in the upper half of the coding-space.

The two empirical groups for which the linear separators did not allow unambiguous assignment were the *unspecific* (light grey) and *few-fast type DINs* (dark grey, Fig 2E). Both of them overlapped with *ON-type velocity-sensitive DINs*. Since *few-fast type DINs* fired more during movement than during hold-phases, they were all positioned in the left half of the coding-space. Accordingly, our data-driven analysis suggests that this group may be considered a sub-group of *ON-type velocity-sensitive DINs*. In case of the *Unspecific DINs*, their predominant location near the origin of the coding-space underscores the appropriateness of the group’s name. As yet, it cannot be assigned automatically.

The good overall correspondence of the empirical grouping and distinct sub-regions of the coding-space suggests that the coding-space could also be used for quantitative comparison of real DINs with modelled DINs (see below).

### Modelling DIN input: Hair fields of an antennal joint

Since the analysis above showed that DINs were mostly sensitive to antennal movement and posture, their presynaptic sensory afferents must encode at least these two variables. Out of the different mechanoreceptors on the insect antenna [2] and of those of stick insects in particular [13], antennal hair fields seemed to be particularly well-suited for mediating the information required. Antennal hair fields have phasic-tonic response properties and an intrinsic velocity sensitivity [20]. Ablation of antennal hair fields affects active antennal movements in stick insects [21], and impairs tactually-induced turning in cockroaches [22]. Therefore, antennal hair fields are involved in the local coordination of antennal joints and the descending control of locomotion. Moreover, afferents from antennal hair fields overlap with DIN dendrites in the brain and gnathal ganglion [23], where they are likely to provide direct input to at least some DINs [17]. Therefore, a major objective of our study was to test how much of the DIN response properties could be explained with antennal hair fields providing their only afferent input.

**Fig 3:**
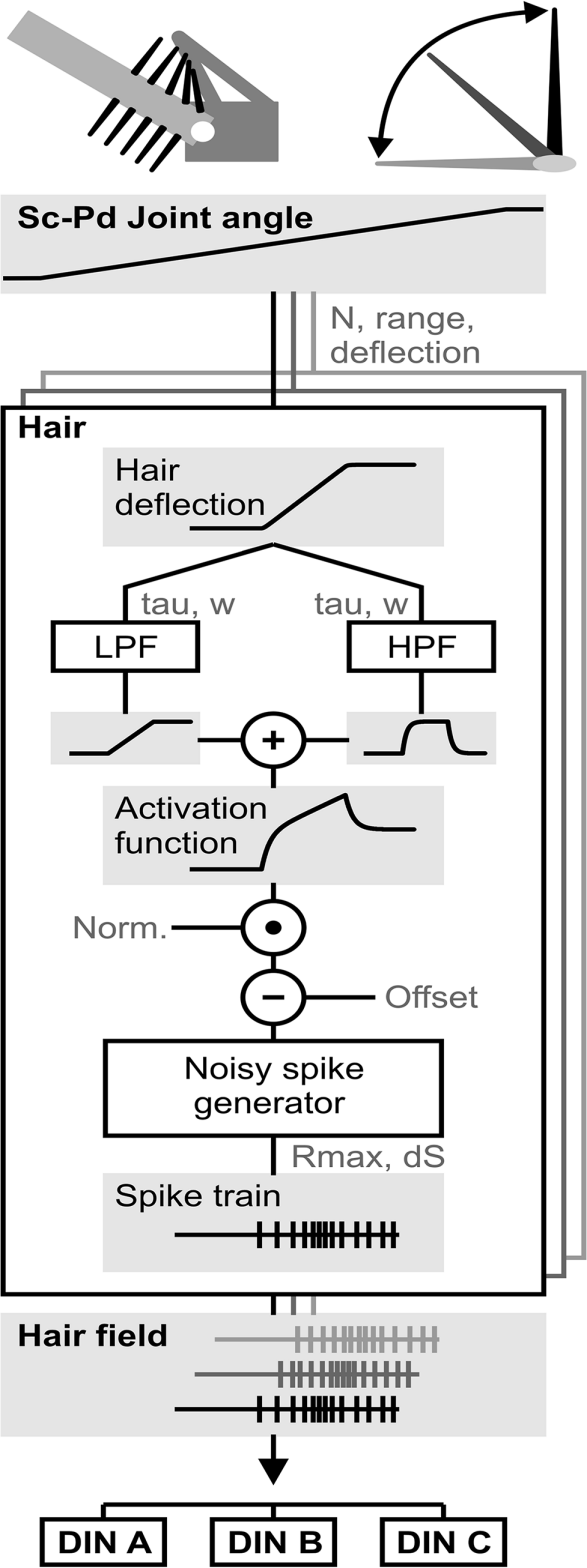
Antennal hair field model. The schematics at the top show the basic concept of the model. Individual sensilla are placed in a hair row along the long axis of the pedicellar base, and are deflected once the flagellum moves. Each sensillum can be deflected (right). The velocity and amplitude of the deflection depends on angle and movement of the Sc-Pd joint. As the Sc-Pd joint angle changes, individual hairs are deflected according to their position within the hair field (Hair deflection). The hair angle is low- and high-pass filtered (LPF, HPF), and the two resulting filtered traces are subsequently added (+). This yields the activation function, which is an approximation of the membrane potential of each hair field afferent. After normalization (norm.) and subtraction of an offset, this activation function is fed into a noisy spike generator (see Material and Methods), which transforms the continuous activation function into discrete spike trains. This is done in parallel for all hair field afferents (grey boxes in background), generating spike trains for all afferents (Hair field). Ultimately, these spike trains are used as the input to a DIN model (see Fig 6). N: number of hairs per hair row; range: sensitivity range of each hair; deflection: characteristic describing the dependence of hair angle on joint angle; LPF: low pass filter; HPF: high pass filter; tau: time constant of linear filter (LPF or HPF); w: weight factor; Offset: constant that is subtracted from the activation function; Rmax: maximal spike rate of the afferent; dS: sampling interval (1 ms). Norm., normalization used to scale the activation function. Grey boxes highlight example time-courses. The parameter settings are given in Table 1. See main text for further details.

As a first step towards this goal, we developed a computational model of antennal hair field afferents to estimate the synaptic input to DINs during Sc-Pd joint movement (Fig 3). The model parameters are listed in the caption to Fig 3, their values are given in Table 1.

**Table 1:** Parameter settings for the hair field model shown in Fig 3.

N (hairs)	Range	Deflect.	LPF (tau/w)	HPF (tau/w)	Offset	Norm.	Rmax	dS
20	2.5°	slope 1	10 ms / 2	30 ms / 20	35	100	300 1/s	0.001 s

Hair fields contain patches or rows of several mechanoreceptive hairs [2]. In stick insects, Sc-Pd joint movement is monitored by two hair fields on the dorsal, and a further two on the ventral surface of the pedicel [21]. As described in detail for the cockroach antenna [20], our model assumes that antennal movement causes the deflection of individual hairs (or sensilla) of antennal hair fields. The further an antennal joint moves away from its resting posture, the more hairs of a hair field are being deflected (Fig 3, schematic on top). Each hair contributes to encoding a fraction of the joint angle range. This range is defined by a minimum and a maximum joint angle, equivalent to the onset of deflection and its saturation. In our model, we collapsed these hair fields into two hair rows, one on each side of the joint (Fig 3, schematic on top). Each hair row consisted of 20 hairs (Table 1), which is a conservative estimate of the average number of hairs in the stick insect [21]. The spacing of hairs was assumed such that the number of deflected hairs varied linearly with antennal joint angle. Dorsal hairs were only deflected in the dorsal joint angle range (Sc-Pd joint angle > 0°), and ventral hairs only in the ventral joint angle range (Sc-Pd joint angle < 0°, Table 1).

For simplicity, the deflection angle of each hair (*hair deflection* in Fig 3) was assumed to vary linearly with the *Sc-Pd joint angle* within the hair’s sensitivity range. The activation of sensory afferents was then calculated by low- (LPF) and highpass-filtering (HPF) the hair angle. The filtered traces are shown in Fig 3 (grey boxes). Subsequently, the two filtered traces were added (Fig 3, +). The resulting *activation function* can be viewed as the hypothetical membrane potential of the sensory afferent. After normalization and subtraction of a constant *Offset*, the activation function was fed into a simple *Noisy spike generator*, as described in the section Material and Methods. The spike generator turned the activation function into a series of discrete spike times. Each model afferent had an absolute refractory period of 3 ms. The same computations were carried out for all hairs of the two hair fields, i.e., for 2x20 mechanosensory afferents (Table 1 and Fig 3). Note that the assumption that the properties of all sensilla were the same is a simplification [20]. The output of the hair field model was a set of *Spike trains*, one for each afferent (Fig 3, Hair field).

**Fig 4:**
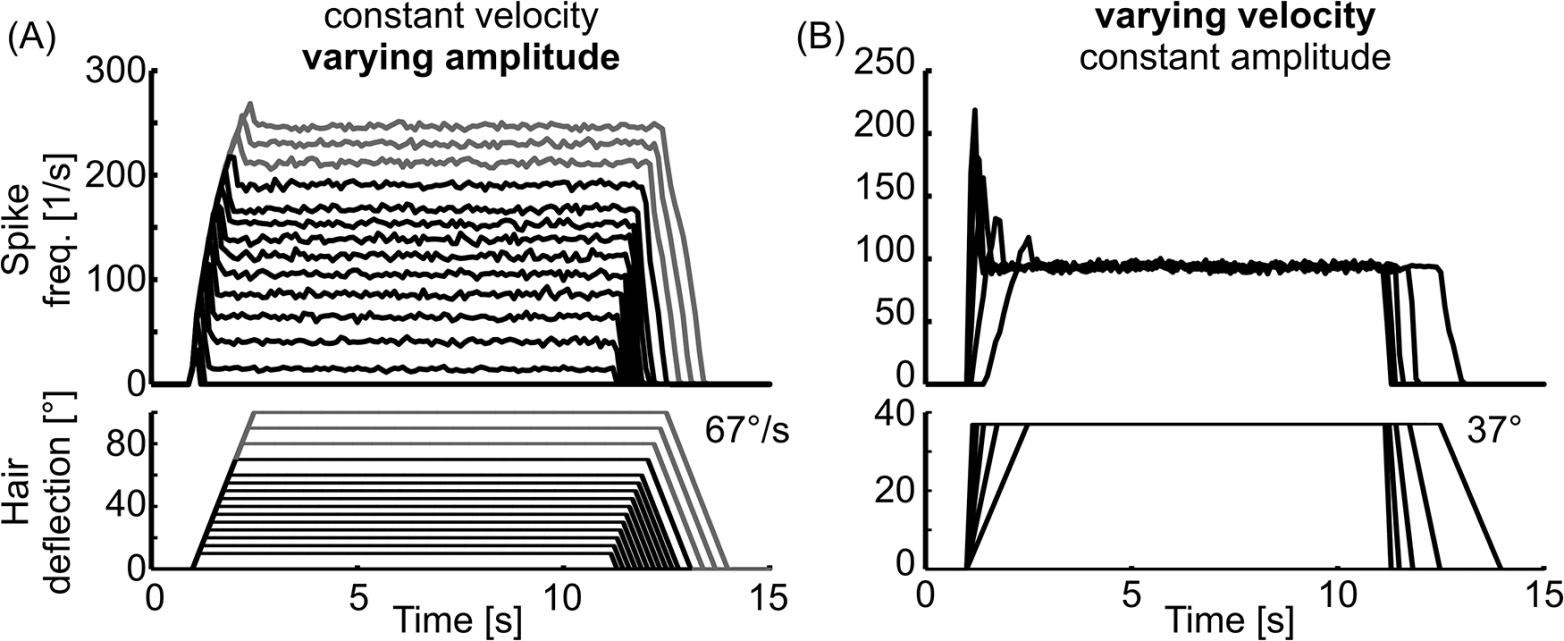
Properties of individual hair field sensilla. The parameters of the model were tuned such that the graphs in A and B match recordings of cockroach antennal hair field sensilla [20]. A) The mean spike rate of the sensillum depended on the deflection angle. Mean spike rate (upper traces) during deflection (lower traces) of a single sensillum. The amplitude of the deflection was varied while the velocity was kept constant. B) The peak spike rate during deflection was proportional to the angular velocity of the deflection, as is the case for cockroach sensilla. Deflection velocities were 25, 50, 90, 150, and 250°/s, the amplitude of deflection was kept constant.

It was possible to tune the parameters of our hair field model such that the properties of output spike trains reflected those measured in hair field afferents of the cockroach antenna (Fig 4, compare to Fig 7 of [20]). Note that the stimulus time courses in Fig 4 (*Hair deflection*) were chosen such that they mimicked those used by [20]. Most importantly, the transient activity of the sensory afferents during ramp-and-hold stimuli was proportional to the deflection velocity (Fig 4A), while the sustained activity was proportional to the deflection amplitude (Fig 4B). Hence, each individual afferent had the same overall response properties as afferents on the cockroach antenna [20].

**Fig 5:**
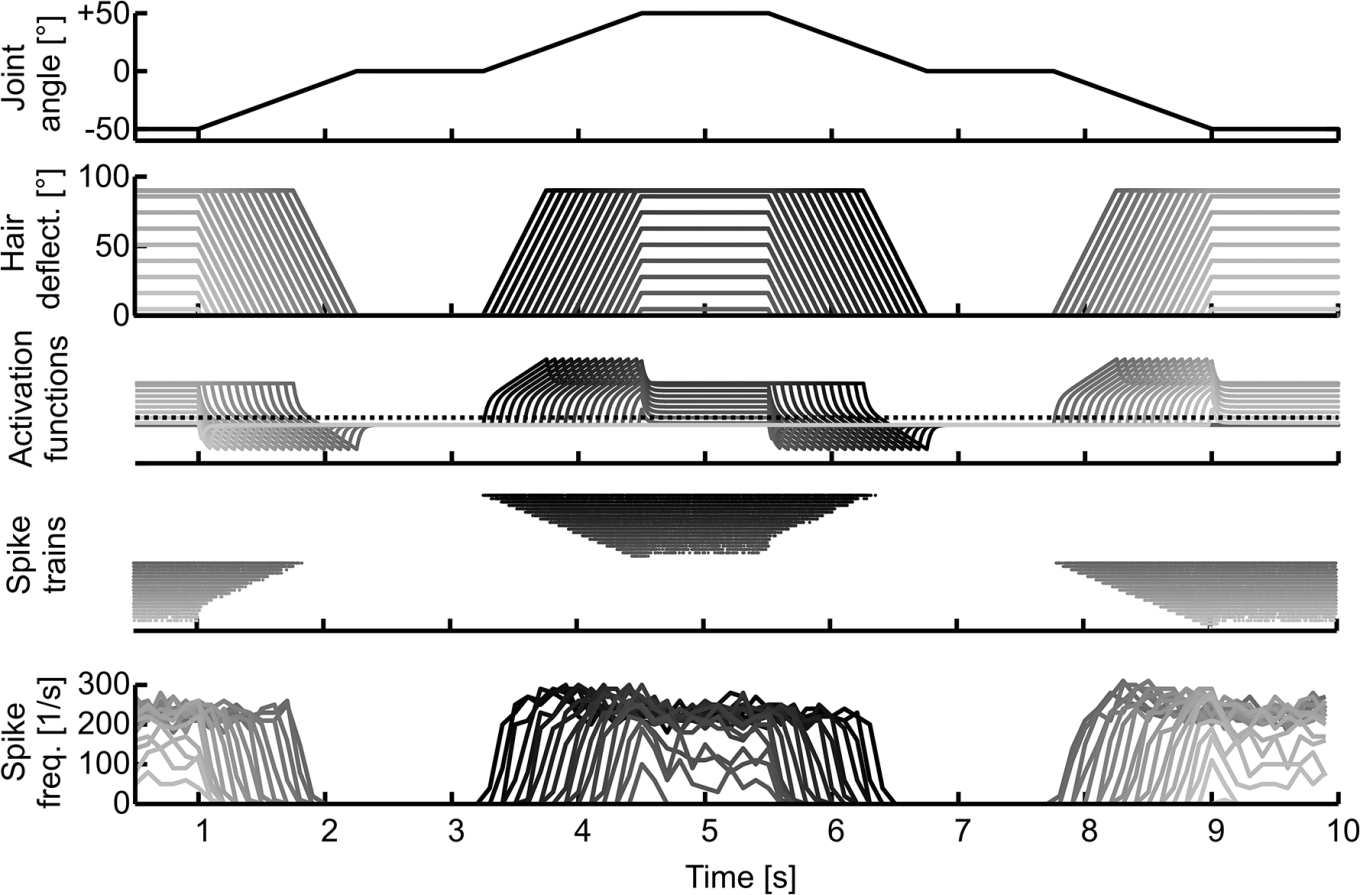
Computational flow of the antennal hair field model illustrated in Fig 3. (input to output displayed from top to bottom). Each plot corresponds to one level of the hair field model. As the antennal joint angle changes (Joint angle), the individual sensilla are deflected according to their position on the pedicel (Hair deflect.). Multiple lines indicate the different sensilla (or their corresponding afferent) of one hair row. Deflection leads to activation of the afferents (Activation functions). The activation functions are used as the input to a simple noisy spike generator, the output of which is a time-varying spike pattern (Spike train). The dotted black line indicates the absolute threshold of the spike generator, which is 0. The instantaneous spike rate (Spike freq.) varies among afferents of the same hair field.

While it is possible to record from individual hair field afferents during deflection of an individual hair field sensillum, it is difficult to estimate the activity of the population of sensory hairs during antennal movement. Therefore, we arranged the modelled afferents in a way that reflected the situation on the stick insect antenna (schematic in Fig 3), and stimulated the hair field model using the same stimulus time course as was used for the experimental characterization of DIN responses (Fig 5, *Joint angle*). As a result, we obtained an estimate of the bulk activity of all hair field afferents during antennal movement (Fig 5, *Spike freq*.). Owing to the different location of individual hairs, movement of the Sc-Pd joint led to distinct time courses of hair deflection (Fig 5, *Hair deflect*.), activation of the corresponding mechanoreceptive afferent (*Activation functions*), and the ensuing spike train elicited in the afferent (*Spike trains*). Hairs of the ventral hair field were deflected in ventral joint angle ranges (Fig 5, light grey), and hairs of the dorsal hair field in dorsal joint angle ranges (Fig 5, dark grey). No hairs were deflected during the stimulus hold phases at the resting posture, i.e., at an Sc-Pd joint angle of 0°. Accordingly, no afferent activity was generated during these hold phases. As the Sc-Pd joint was moved away from this resting posture (in either direction), the sequential deflection of additional hairs caused sequentially delayed activation of the corresponding afferents. Afferents that were activated only at large joint angles did not reach the maximal activation level. This was because the corresponding distal hairs never reached full deflection and, therefore, their low-pass filter contributed less positive activation than in afferents of more proximal hairs. Accordingly, the complete set of afferent spike trains revealed two main differences among afferents: the cascaded onset of spike trains during movement, and the different maximum transient and persistent spike rates for hairs that did not reach full deflection.

### Modeling a diverse population of descending interneurons

The aim of the DIN model was to capture the most important properties of the DIN population and different DIN types as described by [16]. We validated the model by quantifying the differences between real and modelled DINs in the multivariate coding-space. In particular, we tested how much of the diverse DIN properties could be explained by assuming exclusive input from antennal hair field afferents and no other mechanoreceptors. As in the hair field model, we used only first-order linear filters and simple mathematical operations to integrate the afferent hair field activity.

**Fig 6:**
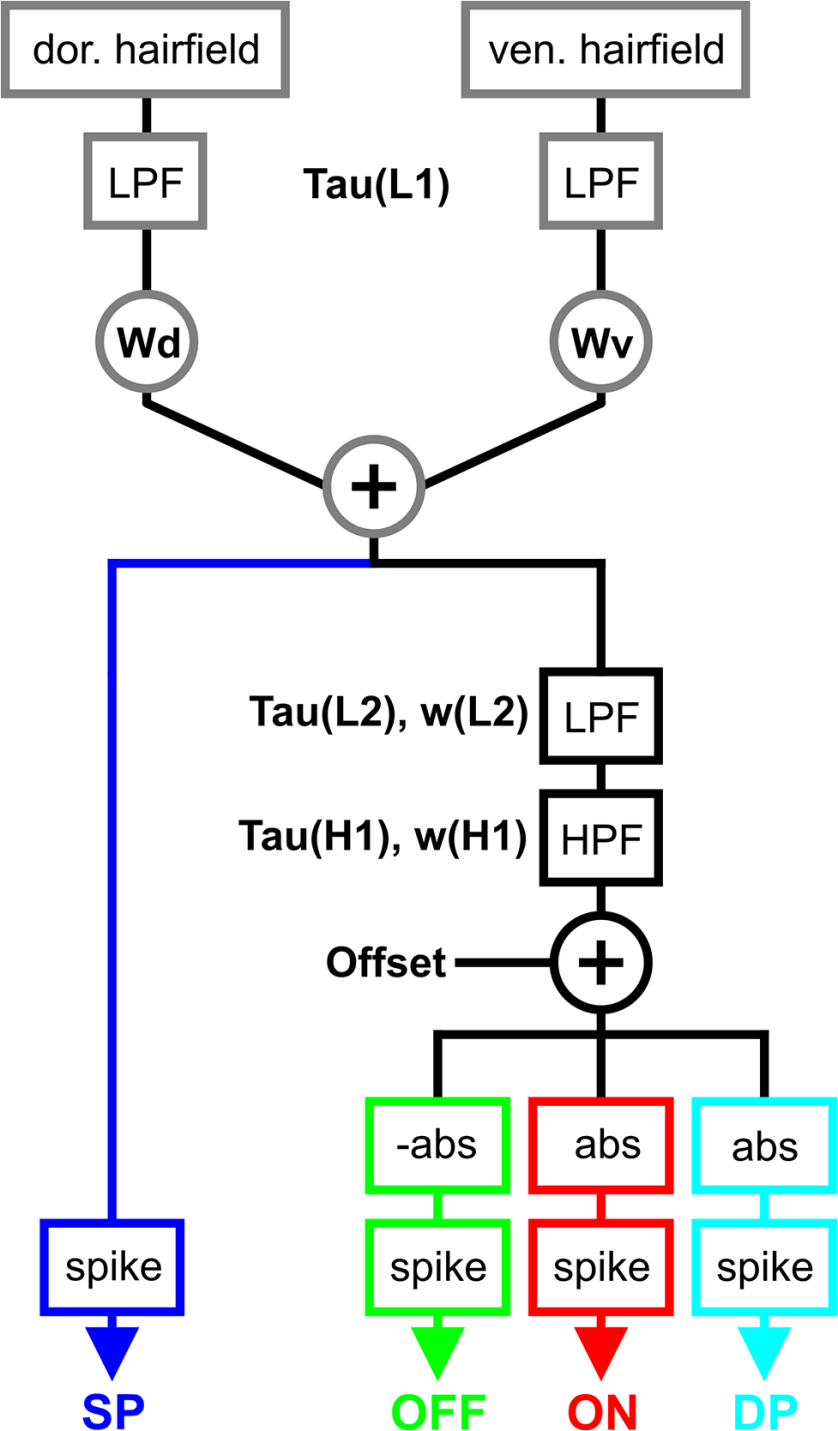
A computational model for different DIN types. Four groups of DINs can be modelled in the same computational framework, using only two modelled hair fields (see Fig 3) as input elements. Modelled DINs integrate afferent spike trains from both hair fields by use of linear low-pass filters (LPF) and weighted summation of the two input streams. In the model variant for *simple position-sensitive DINs*, represented by the left, blue branch, the weighted sum of the two input streams is fed directly into the spike generator (spike). The right branches show the model variant for *OFF-type* (green), *ON-type* (red), and *dynamic position-sensitive DINs* (cyan). This branch contains additional linear filters, subtraction of an offset and a group-specific rectification (abs., see Table 2 and text). Parameters that were varied to model different DIN types are shown in black. Wd: weight of dorsal hair field input; Wv: weight of ventral hair field input; +, addition, Offset: offset used to shift the DIN activation. LPF: low pass filter; HPF: high pass filter. Tau and w describe the time constant and the weight of the respective filters. The parameter settings are given in Table 2.

Four types of DINs were modelled, using slightly differing variants of the same computational framework (Fig 6, different colours represent different DIN types). The first step was common to all model variants (Fig 6, grey). Here, the spike trains of all hair field afferents, i.e., the output of the hair field model (Fig 3), were low-pass filtered separately (LPF, Fig 6). This spread individual spikes in time, and thus generated what may be interpreted as postsynaptic potentials in DIN dendrites. The time constant Tau L1 of this first filtering step was the same for all DIN types (Table 2). The resulting afferent input functions were scaled by separate, adjustable gains for dorsal and ventral afferents (Wd and Wv), and summed (+), yielding the lumped afferent synaptic input to a DIN. This input function was normalized (Table 2) in order to provide a similar activation range to all variants of the DIN model. The resulting input activation level can be considered an abstraction of the membrane potential at the spike-initiating zone.

**Table 2:** Parameter settings for the models of different DIN types shown in Fig 6.

DIN/Param.	LPF 1 (tau)	Wd	Wv	LPF 2 (tau/w)	HPF (tau/w)	Offset	rectify	Norm.	Rmax
**SP v/d**	5ms	0/1	1/0					4	10
**Ex.DP**	5ms	1	1	50/2	40/20	-0.2		20	100
**ON**	5ms	1	1	50/2	40/20	-0.2	abs	20	120
**DP v/d**	5ms	0/1	1/0	50/2	40/20	-0.2	abs	20	100
**OFF**	5ms	1	1	50/2	40/20	+1	-abs	40	30

Param.: parameter; SP: simple position-sensitive DINs; v/d: ventral versus dorsal; Ex.DP: dynamic extreme position-sensitive DINs; ON: ON-type velocity-sensitive DINs; DP: dynamic position-sensitive DINs; OFF: OFF-type velocity-sensitive DINs. All further acronyms as in Figs 3 and 5. Note that rectification was not necessary for simple position-sensitive and dynamic extreme position-sensitive DINs.

### Simple position-sensitive DINs

**Fig 7:**
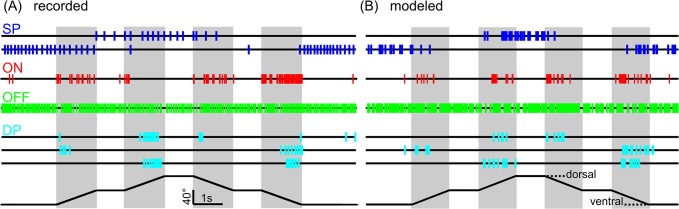
All DIN types could be modelled using only hair field input. A) Examples of spike trains recorded from seven representative DINs during stimulation at movement velocities of 40°/s. Blue, simple position-sensitive DINs (dorsal and ventral); red, ON-type velocity-sensitive DIN; green, OFF-type velocity-sensitive DIN; Cyan, dynamic position-sensitive DINs (dorsal, ventral and extreme position). B) Examples of modelled spike trains evoked by the same stimulus. Colours as in A. The modelled spike trains reflected the overall spike distributions and frequencies of the recorded DINs. Note that OFF-type velocity-sensitive DINs were only weakly inhibited at these relatively slow velocities (see also Fig 8).

The main DIN model was split into two computational branches (Fig 6). The simplest model variant was that of *simple position-sensitive DINs* (Fig 6, blue, SP). This DIN group is characterized by firing in dorsal or ventral joint angle ranges, or both, and by a spike rate that is independent of antennal movement per se [16]. Dorsal or ventral simple position-sensitive DINs could be modelled by summing the activity of all dorsal or ventral hair field afferents, respectively. The input activation level was then directly fed into the random spike generator. The resulting spike time series exhibited similar position dependence as that of real DINs (compare blue spike trains in Fig 7A and 7B).

Varying the normalization of the activation function and the frequency of the spike generator (Table 2) could be used to improve this match. Recorded simple position-sensitive DINs fired more regularly than modelled DINs, because the spike generator used in the model was relatively noisy. We refrained from adjusting the spike-generator in different model variants in order to use as few model parameters as possible, and to thus keep the DIN model as simple as possible. For the same reason, we did not weigh each afferent input separately but used only one gain for all afferents from the same hair field. As a consequence, the tuning of the range boundaries of the position dependence was limited.

As yet, note that there is no need for a spatial map or somatotopy within the hair field sensory system for the derivation of an accurate joint angle signal. The activity of the population of afferents simply increased with an increasing number of deflected hairs, and with stronger deflection of individual hairs (Fig 5).

### Velocity-sensitive DINs

All known ON- and OFF-type velocity-sensitive DINs in the stick insect respond to antennal motion throughout the entire joint angle work range [16]. Note that the two DIN classes termed velocity-sensitive DINs in [16] are not exclusively velocity-sensitive: they are characterised by very strong movement sensitivity and relatively weak posture sensitivity (Fig 2E). Therefore, modelled velocity-sensitive DINs received input from both dorsal and ventral hair fields. Accordingly, the gains associated with these two input streams, Wd and Wv, were set to one (Fig 6 and Table 2). The activity of all afferents was then bandpass-filtered and normalized (Fig 6, green and red branches), to generate transient DIN activity whenever the activity of hair field afferents changed rapidly. As a result, DIN activation was high during Sc-Pd joint movement.

However, upon returning to the resting position, the transients in the DIN activation level turned negative. This occurred when ventral hairs (first stimulus ramp) or dorsal hairs (third stimulus ramp) stopped spiking (see Fig 5). In contrast, real velocity-sensitive DINs showed similar responses during all four stimulus ramps, i.e., whenever the antenna moved (Fig 7A, red and green spike trains, see also [16]). To avoid these negative transients in the DIN activation level, we used a full-wave rectification (Fig 6, abs). Consequently, antennal movement led to depolarization during any antennal movement for ON-type velocity-sensitive DINs, yielding a spike time series similar to that observed in real DINs (Fig 7A and 7B, red). Note that full-wave rectification is not simulating a physiological mechanism per se, but may be considered a simple mathematical operation that could be achieved by more complex physiological processes that may involve interneuron stages (see also Discussion).

Two further details were special about the model variant for *ON-type velocity-sensitive DINs*: First, an offset was subtracted to keep the activation of the DIN below threshold during hold-phases of the stimulus, and thus to account for their low baseline activity [16]. Second, the spike rate of the spike generator was higher than in other model variants, and set to 120 Hz (Table 2).

*OFF-type velocity-sensitive DINs* are characterized by a high baseline spike activity that gets increasingly reduced with increasing movement velocity of the antenna. To account for this property, the activation function as obtained in the ON-type velocity-sensitive DINs was inverted, scaled by a factor of 0.5, and subtracted from the constant offset of 1 (Table 2). The offset accounted for the baseline activity, which was increasingly reduced with increasing afferent activity, i.e., with increasing movement velocity. As a consequence, the models of OFF- and ON-type velocity-sensitive DINs received the same afferent input, but the gain was half as strong in OFF-type DINs and led to either excitatory (*ON-type DINs*) or inhibitory drive (*OFF-type DINs*,-abs in Fig 6, green).

**Fig 8:**
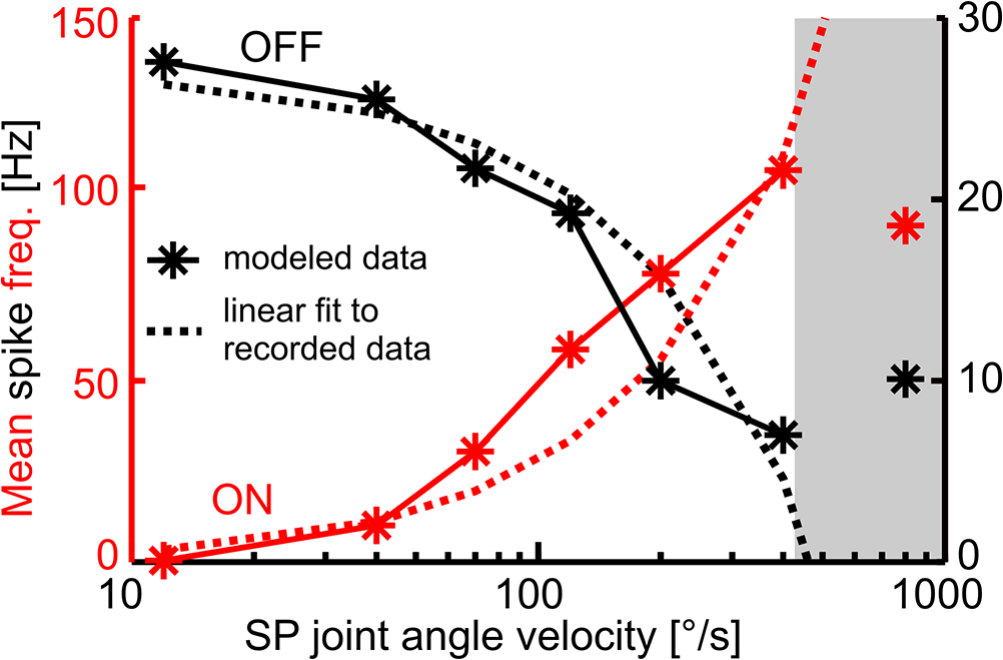
Modelled ON- and OFF-type DINs have a similar velocity tuning as their recorded counterparts. Stars show the mean spike rates of modelled ON-type (red, left y-axis) and OFF-type (black, right y-axis) velocity-sensitive DINs at different velocities. The dotted lines represent linear fits to the mean spike rates of two representative recorded DINs. The curvature of the linear fits results from the semi-logarithmic plotting. Grey area is outside the range used for the PCA. Values are means of n = 4 sweeps.

This model could account for the velocity sensitivity of ON- and OFF-type DINs without tuning the afferent input in a velocity-sensitive manner (Fig 8). Modelled DINs had essentially the same velocity sensitivity as their real counterparts, even when considering a wide range of movement velocities (Fig 8, compare solid and dotted lines). The velocity sensitivity emerged because individual hairs of the hair fields were deflected at higher rates and, more importantly, with decreasing delays between adjacent hairs. This led to higher synchronicity of afferent spikes at higher Sc-Pd joint angle velocities, which in turn resulted in stronger activation of ON-type and stronger inhibition of OFF-type DINs during faster antennal movement. Therefore, the model did not only account for the distribution of spikes during individual stimuli, but also for DIN properties across different stimuli.

As yet, velocity sensitivity broke down for velocities beyond 400/s. This occurred mainly because the impact of filter-induced phase shifts and the resulting latencies of our DIN model were larger during faster movement velocities (Fig 8, grey). Response latencies also affected the analysis of the recorded DIN activity, in that spike rates were underestimated for very fast movements. In order to prevent this effect from confounding data-driven clustering of modelled DIN types and the comparison with real DINs (see below), the responses to the fastest movement stimuli were excluded from the quantitative analysis.

### Dynamic position-sensitive DINs

The fourth model variant concerned *dynamic position-sensitive DINs* (Fig 6, cyan), and was based upon the structure of the variant for ON-type velocity sensitive DINs. *Dynamic position-sensitive DINs* are characterized by a combination of position- and motion-sensitivity, in that they spike only during motion within extremely ventral or dorsal joint angle ranges [16]. By setting the gains Wd (or Wv) to zero (Table 2), the modelled DINs received afferent input from the ventral (or dorsal) hair field only. As a result, motion sensitivity was restricted to a limited range of positions leading to tuning characteristics that are typical for ventral or dorsal specimen of this DIN type (see cyan spike trains in Fig 7A). To account for the fact that spike rates of dynamic position-sensitive DINs were generally lower than those of ON-type velocity-sensitive DINs, the spike rate of the spike generator was reduced to 100 Hz (Table 2).

Dynamic position-sensitive DINs that fire during movement towards both dorsal and ventral extreme positions (see lowest cyan trace in Fig 1) could be modelled by removing the full-wave rectification from the ON-type velocity-sensitive DIN model. As a consequence, the modelled neuron spiked during movement towards the dorsal and towards the ventral extreme position (Fig 7A and 7B, cyan).

In summary, all four important antennal mechanoreceptive DIN groups found in stick insects could be modelled by the same computational framework, with minor differences among the four model variants (Table 2). All response types could be derived from the integration of afferent input from two antennal hair fields only. We did not attempt to model neurons of the *few-fast* and *unspecific movement-sensitive* types, because their spike patterns can be explained by co-stimulation of mechanoreceptors which are not located at the Sc-Pd joint, such as campaniform sensilla.

### Comparing modelled and recorded DINs in the coding-space

To validate our computational modelling framework by means of a quantitative comparison of modelled and recorded DINs, we used the coding-space described above (Fig 2) to compare the model variants to their physiological counterparts. Moreover, we systematically varied model parameters to illustrate their effect on the DINs’ coding properties in a graphical kind of sensitivity analysis.

**Fig 9:**
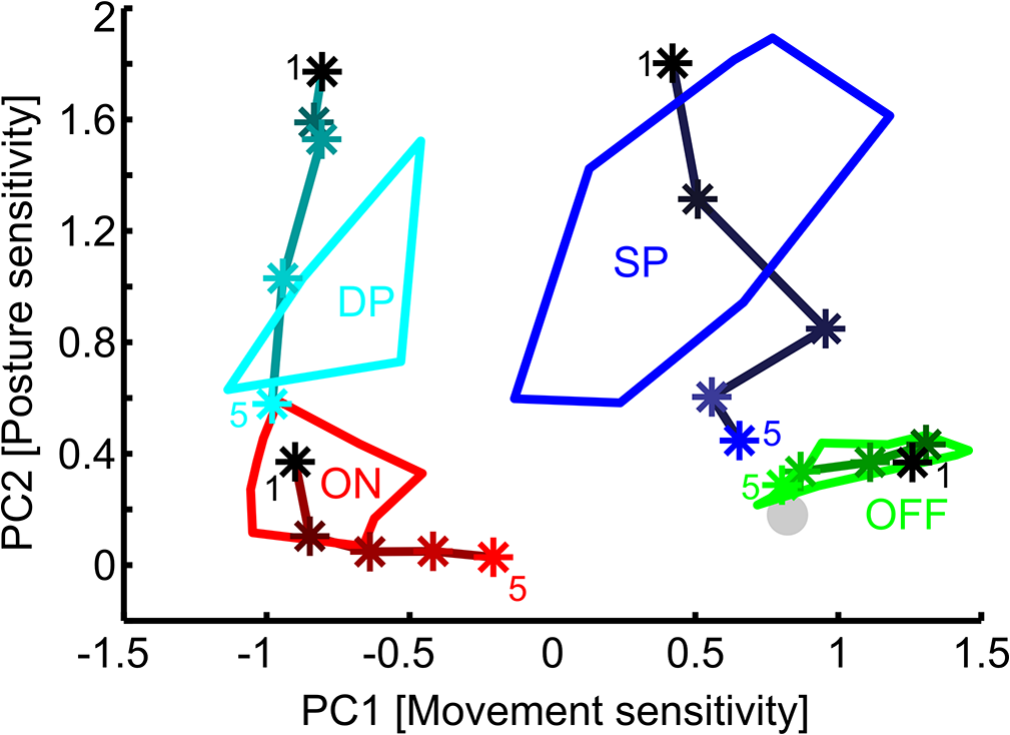
Comparison of modelled and recorded DIN populations in the coding-space. Polygons show the outline of the recorded DIN populations from Fig 2. Cyan: dynamic position-sensitive DINs (DP); red: ON-type velocity-sensitive DINs (ON); green: OFF-type velocity-sensitive DINs (OFF); blue: simple position-sensitive DINs (SP). The y-axis shows the absolute PC 2 value. Asterisks represent the position of different modelled DINs in coding-space. The lines connect DINs of the same model variant. For each variant, only a single model parameter was varied continuously to traverse the range covered by recorded DINs. Parameter variation is indicated by lighter shades of colours with increasing deviation from the original setting (Table 2; black asterisks, labelled 1). The grey circle represents a modelled DIN firing random spikes (i.e. without antennal input). n = 4 sweeps for modelled DINs.

For this, modelled DINs were analyzed exactly like recorded DINs (see Fig 1), and plotted alongside the recorded DINs in the 2D coding-space (Fig 9; compare with Fig 2). Thus, the whole set of model variants, including systematic parameter variation, could be mapped into the same space as the population of recorded antennal mechanosensory DINs.

First, to better define the properties of the coding-space, we modelled a DIN without any antennal input, which fired spikes at random during antennal stimulation (filled grey circle, Fig 9). This DIN ended up close to zero on the y-axis, indicating no posture sensitivity, as expected (Fig 9). However, on the x-axis, which indicates the movement sensitivity, the DIN ended up in the right half of the coding-space. This occured because most DINs in the population fired more during antennal movement than during hold phases. Therefore, DINs exhibiting a spike rate that is independent of antennal movement, i.e., with zero motion-selectivity, end up in the right half of the coding-space, with x = 0.8 indicating no sensitivity to antennal movement in the coding-space.

Modelled and recorded ON-type velocity-sensitive DINs occupied the same region in coding-space (Fig 9, compare red stars and red polygon). Increasing the background activity by increasing the offset of modelled DINs resulted in less movement sensitivity, as illustrated by increasingly light red stars in Fig 9. The offsets used were -0.2 (original model, black star, 1), -0.1, 0, 0.05, and 0.01 (dark to light red, 5). Thus, variation of this single parameter created a trajectory of movement sensitivity in coding-space. This trajectory traversed the range covered by ON-type velocity sensitive DINs (red line, Fig 9) in a curved manner. In general, the modelled DINs of this variant were not posture sensitive because they received symmetric input from dorsal and ventral hair fields (Fig 6). However, there was a tendency for more movement- sensitive neurons to score higher on PC2, too. This was due to the small position component in PC2 (see Fig 2A). Notably, most recorded ON-type velocity-sensitive DINs scored higher on PC2 than modelled DINs, and their position selectivities were often larger than zero, suggesting that they received slightly asymmetric input from dorsal and ventral antennal hair fields.

In the case of *OFF-type velocity-sensitive DINs*, modelled DINs were almost perfectly co-localized with recorded DINs in coding-space (compare green stars and green polygon, Fig 9). OFF-type DINs scored highest on PC1, because their spike patterns were most different from all the other DINs. OFF-type DINs were the only neurons that fired less during movement than hold-phases. In the OFF-type DIN model variant, increasing the weight of the input, i.e., the strength of inhibition during antennal movement, led to a trajectory (green stars) that neatly traversed the narrow range covered by real DINs (green polygon). In the modelled OFF-type DIN in Figs 7 and 8, the normalization was twice as strong as for ON-type DINs. Therefore, the effect of afferent activity on the DIN activity was half as strong as in ON-type DINs. To traverse the complete area covered by recorded OFF-type DINs in the coding-space (Fig 9, green polygon), the normalization value for modelled neurons (Table 2) was varied. We divided the original value (40, black star, original model, see Table 2) by 0.1, 0.4, 0.7, and 1.3. This yielded increasingly sensitive modelled OFF-type DINs because the modelled DINs were increasingly affected by the afferent activity (light to dark green stars, Fig 9). The resultant normalization value of the most-sensitive modelled OFF-type DINs was 40/1.3 = 30.1. This was still 1.5 times the normalization value used for modelling ON-type DINs (20, Table 2). Therefore, even the most sensitive OFF-type DINs were less sensitive to antennal movement than an average ON-type DIN. Similar to ON-type DINs, OFF-type DINs that were more sensitive to antennal motion scored higher on PC2, albeit with different sign (Fig 9). The least sensitive recorded OFF-type DINs overlapped with a modelled DIN that was not sensitive to antennal movement at all (filled grey circle). However, these OFF-type DINs were inhibited at very high movement velocities outside the range used for the quantitative analysis.

To cover the range of posture sensitivity found in real DINs, modelled position-sensitive DINs either received input from only one hair field (black stars at the top, 1), or increasingly strong input from the second hair field as well (light blue/cyan stars, 5). For both *dynamic* and *simple position-sensitive DINs*, the Wd:Wv proportion (Table 2) was varied (1:0, 1:0.2, 1:0.4, 1:0.6, and 1:0.8) to account for their distribution along the position axis in coding-space (PC 2). Modelled dynamic position-sensitive DINs covered the same region as their recorded counterparts. In terms of the motion-sensitivity, the range was similar to that covered by ON-type velocity-sensitive DINs (Compare red and light blue stars, Fig 9). It was possible to increase the spread of the position-sensitive DINs on the x-axis by changing the offset, similar to ON-type position-sensitive DINs. Since the selectivity scores were not well suited to distinguish between dynamic extreme position-sensitive DINs and other dynamic position-sensitive DINs (see description of Fig 1), modelled dynamic extreme position-sensitive DINs were excluded from the PCA comparison.

Modelled simple position-sensitive DINs covered the same region in the coding space as recorded simple position-sensitive DINs (dark blue, Fig 9). They were slightly shifted towards higher movement sensitivity compared to the modelled DIN with no motion- or position-selectivity (filled grey circle, Fig 9). Because of the low spike rates of simple position-sensitive DINs, the movement sensitivity varied somewhat due to trial-by-trial variability (dark blue trajectory, Fig 9). This again reflected the relatively large variability of the movement-sensitivity in the population of recorded simple position-sensitive DINs (dark blue polygon, Fig 9). Some of these DINs fired only five to ten spikes per second while the antenna was held in the dorsal or ventral extreme position. Therefore, the presence or absence of even a single spike during antennal movement had a relatively large effect on the calculated motion sensitivity. Simple position-sensitive DINs were nonetheless clearly separated from all other DIN types in the coding-space, because they were clearly less movement-sensitive than dynamic position-sensitive DINs, and more posture-sensitive than OFF-type velocity-sensitive DINs.

In summary, we modelled different DIN types that matched the properties of recorded DINs in terms of the spike patterns and the sensitivity to antennal posture and movement. Notably, the posture sensitivity and the movement sensitivity of DINs could be accounted for when considering only antennal hair fields as the input mechanoreceptors. Therefore, the largest fraction of the coding properties of the DIN population recorded by [16] could be explained without including any of the other known mechanoreceptors of the antenna.

## Discussion

The modelling approach presented in this report represents four important steps in understanding descending neural pathways involved in active tactile sensing and the control of adaptive locomotion.

### 1) A framework for the quantitative analysis of descending interneurons

Our data-driven analysis used a small set of contrast measures to quantify the selectivity scores of 59 DINs. The scores concerned movement *versus* rest, movement direction, and position. These simple measures were chosen to account equally well for a large variety of DIN tuning curves instead of reflecting the properties of a subgroup of DINs with high fidelity. For example, the analysis was sensitive to the difference between dynamic and simple position-sensitive DINs, but was not suited to differentiate between dynamic extreme position-sensitive DINs and other dynamic position-sensitive DINs. For that, it would have been necessary to include further selectivity scores, such as relative direction.

As the selectivity scores we used are intuitively understandable, the PCA results are interpretable in physiological terms. Another advantage of our selectivity scores is that they can also be applied to active movements. This is possible because the mean spike rates used to calculate the scores are largely independent of the duration of movement or hold phases. Hence, the selectivity scores are also suitable for application to more erratic, asymmetric movements and random hold periods, as expected during active antennal movement [12,21]. Thus, it will be possible to compare DIN responses during active and passive movements of the antennal joints, using the same measures, in the coding-space. Moreover, the same data-driven analysis could be used to characterize and compare antennal mechanosensory DINs across species [7], or across different regions of the central nervous system [24].

In the coding-space, it was possible to use simple, linear thresholds to assign DINs to different subgroups, which supported our initial empirical grouping [16]. This opens the door to easily sort DINs recorded in different contexts. For example, spike trains obtained from extracellular multi-electrode array recordings (e.g., see [24]) could thus be linked to intracellular recordings. The data-driven analysis and the coding-space presented here will therefore be useful for a deeper analysis of different mechanosensory DIN populations.

### 2) Antennal hair field input is sufficient to explain the activity of several distinct classes of descending interneurons

Despite the fact that insect antennae carry different types of mechanoreceptors [2], our model included only antennal hair field afferents. By focussing on hair fields, we tested whether their input would be sufficient to drive different DIN types. The modelled sensory hairs were tuned to reflect the properties of antennal hair field afferents recorded in the cockroach [20]. Using a lead-lag system with parallel first-order high- and low-pass filters, it was possible to model spike trains that greatly resembled the recorded afferent spike trains. Our model is therefore grounded on physiological data. An earlier model of hair fields at a stick insect leg joint focused on the timing of hair deflection and the role of spike rate adaptation for the faithful encoding of the joint angle in presumptive follower neurons [25]. In contrast to this study, we had the advantage of knowing the properties of follower neurons, so that we could directly test whether our hair field model can explain the activity of postsynaptic descending interneurons in the CNS. It was sufficient to use only two hair rows, one dorsal and one ventral, to drive the activity of all important characterized DIN groups. Thus, we proved that hair field input is sufficient for explaining the activity of downstream neurons. Moreover, we showed that hair fields cannot only account for positional responses of downstream neurons, but also for their motion selectivity and velocity tuning. Of course, this is no proof that no other mechanoreceptors are involved in shaping DIN responses, too. Indeed, [7] presented evidence suggesting that antennal campaniform sensilla are involved in encoding antennal movement in crickets. Nevertheless, the high descriptive power of our model suggests that no essential mechanoreceptive input components have been omitted. This is even more important to note when considering two further simplifications of our model: First, we reduced the complexity of the hair field arrays at the stick insect Sc-Pd joint from two hair fields and two hair rows [21] to two hair rows, only. Second, we assumed that all afferents have the same properties. In general, afferents can have different response properties within a single hair field, but most examples presented in the literature are phasic-tonic, and thus have similar properties as our model afferents. Our hair field model could therefore be generalized to other insect limb joints, too.

In summary, we presented the first computational model of an insect hair field with relation to its role in driving the activity of downstream interneurons. We showed that hair field activity can be used to derive an appropriate position signal, but also to derive information about joint movement and the velocity thereof.

### 3) Model variants capture the properties of an entire population of neurons

The proposed computational framework contains relatively few parameters in four variants to explain the response properties of a population of DINs.

Modelled DINs reflected the population of recorded DINs well. In particular, the spike patterns of different simple and dynamic position-sensitive DINs, as well as ON- and OFF-type velocity-sensitive DINs could be modelled by simple integration of hair field spike trains (Fig 7). More complex properties like the velocity sensitivity of ON- and OFF-type DINs were captured by second order integration and band-pass filtering of the hair field activity (Fig 8). Finally, our data-driven analysis revealed a large degree of overlap between the two populations in the coding-space (Fig 9), further validating our DIN model. Moreover, by systematic variation of particular model parameters, we were able to account for the spread of recorded DINs in coding-space and, hence, for the properties of individual DINs belonging to the different groups (Fig 9). Our DIN model thus accurately represents the properties of the different DIN types.

To generate response patterns of position-sensitive DINs, the afferent spike trains were low-pass filtered and summed up, which can be interpreted as an abstraction of spatially summated EPSPs. It is conceivable that real DINs receive input from different sensory hairs. Subsequently, the input could be summed up at the spike-initiating zone to elicit spike trains. In such a model, DINs would get direct input from hair field afferents, without the need for an additional interneuronal integration step.

Notably, it was not necessary to distinguish between individual afferents or to weigh their contribution according to their position in the hair field. Hence, our model shows that there is no need for labelled lines or somatotopy in this sensory system. Instead, simple summation of hair field activity is sufficient to explain the coding properties of position-sensitive DINs. In contrast, the position of antennal contacts along the flagellum was proposed to be encoded by a labelled-line principle [26].

Extracting dynamic information from the hair field activity was more complicated. First, it was necessary to add a band-pass filter to extract transients in the afferent activity during Sc-Pd joint movement (Fig 6). A neural correlate of this could be fast adaptation of the DINs, so that only the initial afferent spikes lead to DIN spikes. After band-pass filtering, the onset of afferent spiking during movement away from the resting position leads to an increase in DIN activation. However, the offset of afferent spiking during movement towards the resting position leads to a decrease. Hence, it was necessary to rectify the DIN activation in order to generate similar DIN responses during movement in both directions at the same joint angles.

**Fig 10:**
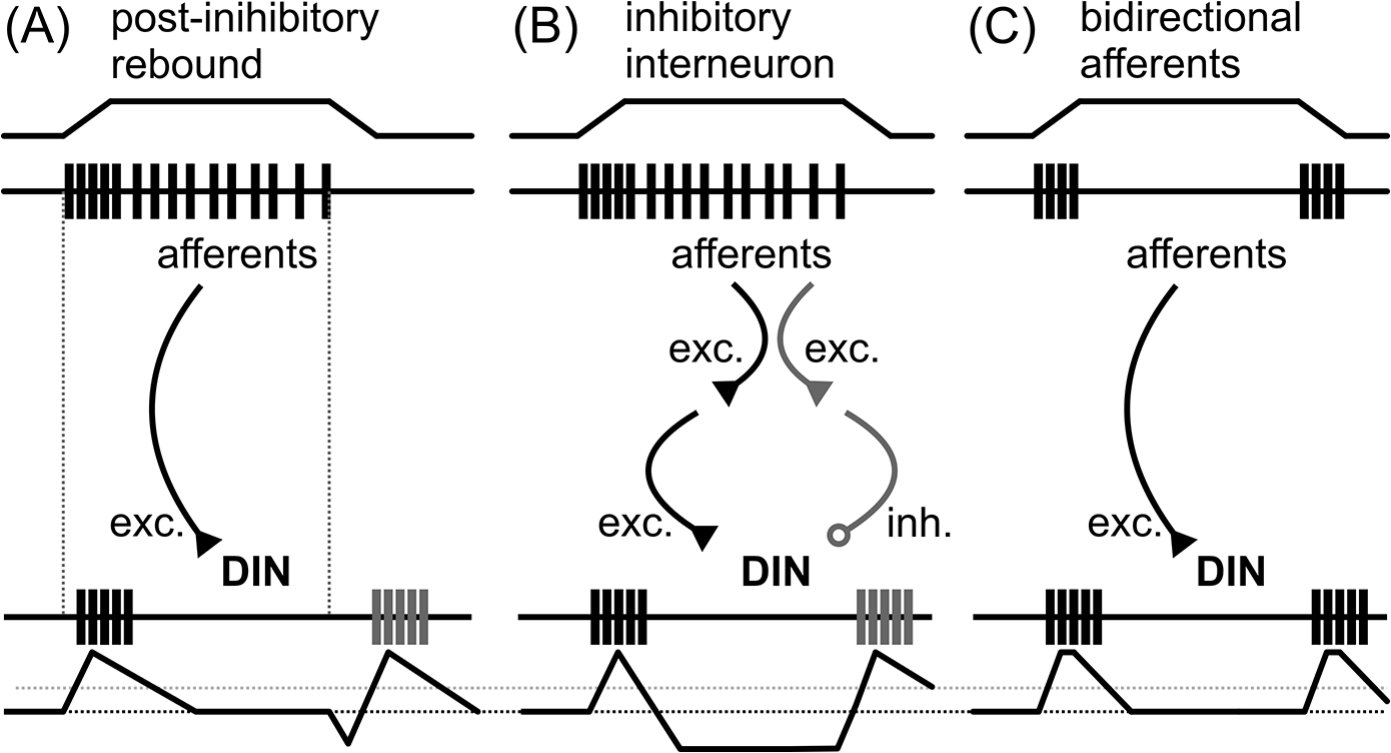
Possible mechanisms underlying the response of ON-type velocity-sensitive DINs. The upper traces represent deflection and return of a sensory hair. The upper spike train represents spiking of the sensory afferent, and the lower spike train represents the DIN’s spike response, given the network in the middle. Black lines with triangles represent excitatory (exc.), and grey lines with open circles inhibitory synapses (inh.). The lowest traces represent putative DIN membrane potentials, the black horizontal dotted lines indicate a membrane potential of zero, the grey dotted horizontal line indicates the spike threshold. A) A direct connection between afferents and DINs could lead to a delayed response after postinhibitory rebound during stimulus ramps 1 and 3. Vertical dotted lines indicate the different delays. B) Parallel excitatory and inhibitory connections between afferents and DINs could lead to a symmetric response during all stimulus ramps, i.e., whenever the antenna moved. C) Additional afferents with bidirectional phasic responses could directly drive DINs with the recorded properties.

The implementation of such a full-wave rectification is not straight-forward to explain in a real neuron. Several mechanisms are possible. First, DINs could have post-inhibitory rebound properties that might lead to the initiation of spikes after hyperpolarisation (Fig 10A; [27,28]). In that case, one should expect that spikes generated by excitatory input would occur faster than rebound spikes, which usually have relatively long delays [29]. Hence, DIN spikes during stimulus ramps one and three would be expected to occur later than spikes during stimulus ramps two and four (Fig 10A, vertical dotted lines). Such systematic differences were not present in velocity-sensitive DINs [16]. Therefore, a rebound mechanism is an unlikely explanation.

Second, the DIN response could be explained by a polysynaptic pathway (Fig 10B). Afferents could drive an interneuron that inhibits a fast-adapting DIN. In such a network, stop of afferent activity would lead to a rapid decrease in the spike rate of the interneuron which, in turn would release the DIN from inhibition. This would have to happen only during movement towards the resting position, when dorsal or ventral hairs return to their initial position (Fig 10B). The appeal of this model is that ON-type velocity-sensitive DINs have a very low baseline activity, which could be due to strong inhibition. However, hair field afferents and ON-type velocity-sensitive DINs overlap morphologically in the gnathal ganglion (or suboesophageal ganglion), suggesting they could be directly connected. This is also supported by the short delays of the velocity-sensitive DIN responses, which forward spikes to the thorax within 11 ms after antennal stimulation [16, 17]. Nonetheless, it is possible that an interneuron might be interspersed between antennal mechanosensory afferents and DINs.

Third, the DIN could receive input from antennal mechanoreceptors with bidirectional phasic properties (Fig 10C). These would be well-suited to make direct synaptic contacts with DINs. For example, the activity of dynamic position-sensitive, ON-type, and OFF-type velocity-sensitive DINs could be derived by simple summation of the activity of bidirectional, phasic sensory hairs that fire both during deflection away from and towards their resting position. Type 1 afferents in the cockroach trochanteral hair field have exactly these properties [19]. It is either possible that the stick insect antenna carries hairs with similar response properties, or that other mechanoreceptors, like campaniform sensilla [30] or chordotonal organs [31], provide this kind of input.

Which one of the above options (Fig 10) may be implemented can be tested in future experiments, for example by measuring the effect of hair field ablation on the DIN spike patterns.

### 4) Model predictions about sizes of DIN ensembles

The quantitative analysis shown in Fig 9 allows us to draw conclusions about the DIN population that otherwise would be difficult to justify. For example, the distribution of DINs in the coding-space and the number of parameters needed to explain the spread of modelled DINs within the coding-space permits the derivation of hypotheses concerning the number of individual DINs belonging to the different clusters. All recorded OFF-type DINs formed a continuous cluster and could be modelled by the gradual variation of a single parameter, the input strength or normalization. This suggests that that there are only few individual OFF-type DINs per antenna. This is also supported by the DIN morphology because, so far, only a single morphological type of OFF-type DIN was identified [17]. Assuming only a single OFF-type DIN on each side of the CNS, the sensitivity of the DIN could either differ between animals, or with dependence on the animal’s state during the recording. It was not possible to model the different sensitivities of OFF-type DINs by changing other parameters, like the baseline spike rate, which differed across animals in the recordings [16].

A similar argument can be made for ON-type velocity-sensitive DINs. The dense distribution of these DINs in the coding-space and the possibility to model them well by shifting a single parameter suggests that only few individual DINs belong to this DIN cluster. In fact, physiological data suggest that only two individual DINs with very similar properties belong to this cluster. The two DINs mainly differ in that one responds to the ipsi-, and the other to the contralateral antenna [17].

The widely varying sensitivity values of both simple and dynamic position-sensitive DINs, on the other hand, and the fact that both posture sensitivity and movement sensitivity needed to be varied to capture the variance in the distributions of these DIN types in the coding-space suggest the presence of a larger number of individual neurons. This is also supported by strongly varying response patterns when comparing different individual recordings [16].

In summary, the quantitative analysis and the DIN model support our findings that few individual DINs belong to the velocity-sensitive DIN clusters. Moreover, the modelling approach suggests that many individual DINs belong to the position-sensitive DIN clusters. Hence, we assume that antennal movement velocity is encoded by a small number of broadly-tuned DINs, while antennal position is encoded by a large number of DINs with variable tuning characteristics. The latter was also suggested by [7] for antennal mechanosensory DINs in the cricket.

While the neural substrate of the connection between DINs and thoracic inter- and motorneurons has not yet been unravelled, the modelled descending pathway is likely to contribute to the intersegmental spatial coordination of antennae and legs in a reach-to-grasp paradigm [5]. As such, it might well represent coding principles in sensory-guided locomotion in general, and inter-limb coordination in particular. Given the detailed knowledge about antenna-driven behaviour and thoracic motor networks in the stick insect, our model of the pathway comprising antennal mechanoreceptors and descending interneurons may be linked to models of thoracic neural networks (e.g., [32,33]) or more abstract, behaviour-based single-leg controllers (e.g., [34,35]). This will pave the way for a better understanding of descending influences on thoracic motor networks, as our model bridges the gap between two well-characterized systems.
